# Periodontal and Peri-Implant Microbiome Dysbiosis Is Associated With Alterations in the Microbial Community Structure and Local Stability

**DOI:** 10.3389/fmicb.2021.785191

**Published:** 2022-01-25

**Authors:** Yuchen Zhang, Yinhu Li, Yuguang Yang, Yiqing Wang, Xiao Cao, Yu Jin, Yue Xu, Shuai Cheng Li, Qin Zhou

**Affiliations:** ^1^Key Laboratory of Shaanxi Province for Craniofacial Precision Medicine Research, College of Stomatology, Xi’an Jiaotong University, Xi’an, China; ^2^Department of Implant Dentistry, College of Stomatology, Xi’an Jiaotong University, Xi’an, China; ^3^Shenzhen-Hong Kong Institute of Brain Science-Shenzhen Fundamental Research Institutions, The Brain Cognition and Brain Disease Institute, Shenzhen Institutes of Advanced Technology, Chinese Academy of Sciences, Shenzhen, China; ^4^Department of Advanced Manufacturing and Robotics, College of Engineering, Peking University, Beijing, China; ^5^Department of Prosthodontics, School and Hospital of Stomatology, Peking University, Beijing, China; ^6^Department of General Dentistry and Emergency Room, College of Stomatology, Xi’an Jiaotong University, Xi’an, China; ^7^Department of Computer Science, City University of Hong Kong, Kowloon, Hong Kong SAR, China

**Keywords:** periodontitis, peri-implantitis, microbiome, community structure, metagenomic sequencing, dysbiosis, local stability

## Abstract

Periodontitis and peri-implantitis are common biofilm-mediated infectious diseases affecting teeth and dental implants and have been considered to be initiated with microbial dysbiosis. To further understand the essence of oral microbiome dysbiosis in terms of bacterial interactions, community structure, and microbial stability, we analyzed 64 plaque samples from 34 participants with teeth or implants under different health conditions using metagenomic sequencing. After taxonomical annotation, we computed the inter-species correlations, analyzed the bacterial community structure, and calculated the microbial stability in supra- and subgingival plaques from hosts with different health conditions. The results showed that when inflammation arose, the subgingival communities became less connective and competitive with fewer hub species. In contrast, the supragingival communities tended to be more connective and competitive with an increased number of hub species. Besides, periodontitis and peri-implantitis were associated with significantly increased microbial stability in subgingival microbiome. These findings indicated that the periodontal and peri-implant dysbiosis is associated with aberrant alterations in the bacterial correlations, community structures, and local stability. The highly connected hub species, as well as the major contributing species of negative correlations, should also be given more concern in future studies.

## Introduction

Periodontitis is a prevalent disease in the human oral cavity and the major cause of dentition defects (Albandar, 2005). It is a complex infectious disease resulting from infection-induced inflammation and hyperimmune response toward various microbial pathogens (Kajiya et al., 2010; Bueno et al., 2015). Previous studies have proved that periodontitis is initiated with microbial dysbiosis in the periodontium (Kinane et al., 2017). The prevalence of periodontitis is estimated from 4 to 76.0% in developed countries and from over 50% to almost 90% in developing ones (Jiao et al., 2021). Approximately over 700 million adults are suffering from periodontitis worldwide (Kassebaum et al., 2014), which has become a severe burden in the oral health of humankind (Marcenes et al., 2013).

Peri-implantitis has been described as a pathological condition around dental implants where inflammation continuously affects connective tissue and finally leads to the loss of the supporting bone matrix (Schwarz et al., 2018). Similar to periodontitis, peri-implantitis is also caused by the hyper-inflammation in peri-implant tissue and the aberrant change in the microbial community (Alcoforado et al., 1991; Leonhardt et al., 1999; Wang et al., 2016). A meta-analysis in 2017 indicated that the weighted mean prevalence of peri-implantitis was around 19.83% at patient level (Lee et al., 2017). As implant-supported prostheses are being more and more widely used to replace missing teeth (Buser et al., 2017), there will be an increasing number of patients suffering from peri-implantitis in the coming future.

Periodontitis and peri-implantitis share many clinical and etiological features, including biofilm-mediated infection, hyperinflammatory reaction, and progressive absorption of alveolar bone (Berglundh et al., 2011; Carcuac and Berglundh, 2014; Liu et al., 2020). Most importantly, the accumulation of dental plaque and the following microbial dysbiosis are considered to be the initiation of both diseases (Ng et al., 2021). Given the shared nature as infectious diseases between periodontitis and peri-implantitis, it is necessary to delve into the microbial communities around teeth and implants to understand the two diseases further.

The stability of commensal microbial communities in human bodies has been proved essential to human health (Relman, 2012). However, previous studies investigating oral microbiota using high-throughput sequencing approaches have mainly focused on the taxonomical profile or microbial functionalities (Dabdoub et al., 2016; Ai et al., 2017; Babaev et al., 2017; Belstrom et al., 2017; Ghensi et al., 2020; Komatsu et al., 2020; Ng et al., 2021). Yet, the community structure and the microbial stability have not been fully illustrated, especially when the complexity of numerous bacterial correlations cannot be fully identified by isolating pairwise interactions. To fill this insufficiency, we analyzed 64 microbial samples from plaque around teeth and implants in different health conditions using metagenomic shotgun sequencing. We annotated taxonomical information at the species level, visualized the bacterial co-occurrence network, analyzed the community structure, and calculated the microbial stability of our samples to further our understanding of periodontitis and peri-implantitis.

## Materials and Methods

### Participant Recruitment

This study enrolled 34 participants, including 19 subjects for the healthy group and 15 subjects with periodontitis or peri-implantitis for the diseased group (See Supplementary Tables 1, 2). All participants were Chinese natives who sought care at the College of Stomatology, Xi’an Jiaotong University, and provided written consents. Natural teeth were considered periodontal health when there was no bleeding on probing (BOP), no clinical attachment loss (CAL), or radiographic bone loss (RBL) and the maximum probing depth (PD) was less than 3 mm. Periodontitis was diagnosed with an increased PD of more than 4 mm, examinable RBL, and interdental CAL, which corresponded with the latest diagnostic criteria for Stage II-IV periodontitis (Papapanou et al., 2018). As for implants, subjects were considered peri-implant health when peri-implant tissue showed no redness, suppuration, BOP, and no more than 1-mm marginal RBL beyond bone remodeling. Peri-implantitis was diagnosed when there was clinical inflammation, increased PD of more than 6 mm, and radiographic evidence of more than 3 mm RBL compared to baselines (Lindhe et al., 2008). Detailed inclusion and exclusion criteria are listed in Table 1.

**TABLE 1:** Detailed inclusion and exclusion criteria for subject recruitment.

Type	Health condition	Inclusion criteria	Exclusion criteria
Teeth	Periodontal health	• Individual normal occlusion with no less than 28 teeth left in dentition; • No RBL or examinable CAL; • Maximum PD ≤ 3 mm; • No BOP or redness examined.	• Diabetes mellitus or other severe systemic diseases; • HIV infection or other severe immune diseases; • A history of tobacco smoking; • A history of immunosuppressant therapy; • A history of bisphosphonates, steroids, or other therapy influencing bone metabolism; • Antibiotic therapy, oral antiseptic therapy, or oral prophylactic treatment undergoing or in recent 3 months; • Having other dentures in any form besides the selected dental implant; • Pregnancy or lactation; • Over 60 years old or below 20 years old.
	Periodontitis	• Individual normal occlusion with no less than 20 teeth left in dentition; • Examinable interdental CAL ≥ 3 mm; • PD ≥ 4mm; • Examinable RBL; • Existing BOP and/or suppuration.	
Implants	Peri-implant health	• A single implant with a single cement-retained crown seated to replace the missing tooth; • Implant in function for over 2 years; • Radiographic MBL ≤ 1 mm; • No redness, suppuration, or BOP examined around the implant.	
	Peri-implantitis	• A single bone-level implant with a single cement-retained crown seated to replace the missing tooth; • Implant in function for over 2 years; Radiographic MBL ≥ 3 mm compared to baseline; • PD ≥ 6 mm around the implant.	

### Clinical Examination and Sample Collection

Before sampling, full-mouth examinations were conducted on all subjects by the same calibrated clinician to record clinical and demographic features, including sex, age, PD, BOP, and RBL. Especially for subjects with implants, we also recorded their implant type, location, and functional time (Supplementary Tables 1, 2).

The selection of sampling sites followed the criteria in our Supplementary Information. When sampling commenced, patients first gargled with distilled water for 1 min. Then, we used cotton rolls to isolate the selected sites and sampled the supragingival plaque using sterile curettes by a single horizontal stroke on each site. Bacteria were washed off from the curettes by rinsing in 1.5-ml microcentrifuge tubes containing phosphate-buffered saline (PBS). The remaining supragingival plaque was then removed. Afterward, we used sterile endodontic paper points for subgingival sampling (Jervoe-Storm et al., 2007), by inserting paper points as deep as possible into the periodontal or peri-implant sulcus and staying for 20 seconds. After taking out, paper points were transferred into 1.5-ml microcentrifuge tubes containing PBS. All samples were stored at −80°C and were then sent to BGI Institute (BGI Group, Shenzhen, China) for genomic DNA extraction, metagenomic libraries preparation, and sequencing.

### DNA Extraction and Metagenomic Sequencing

Genomic DNA of the samples was isolated using QIAmp DNA Micro Kit (Qiagen, Valencia, CA) with “Protocol: Isolation of Genomic DNA from Tissues” according to the handbook. The sequencing libraries were then prepared following BGI’s instruction (BGI Group, Shenzhen, China). The libraries were sequenced on the BGI SEQ-500 sequencing platform (BGI Group, Shenzhen, China). Raw reads generated from the sequencing platform were then filtered and cleaned before further analysis.

### Metagenomic Analysis

To obtain high-quality data, we firstly filtered the raw reads when they contained more than 10 low-quality bases (< Q20) or 15 bases of adapter sequences with a self-constructed script. Using BWA software (version 0.7.17), we aligned the read data to the human genome (hg19) and filtered the reads when the alignment length exceeds 40% of the read length (Li and Durbin, 2009). After the removal of host mapped reads, the clean metagenomic data were applied for the following metagenomic analysis.

Using MetaPhlAn3 (Truong et al., 2015), we aligned the filtered reads to the microbial database of specific marker genes (mpa_v30_CHOCOPhlAn_201901) and obtained the taxonomical annotation results. Based on the microbial profiling, we calculated the relative abundances of bacteria at phylum, class, order, family, genus, and species levels, respectively (see Supplementary Data Sheet 1). After the taxonomical annotation, we performed permutational multivariate analysis of variance (PERMANOVA) to evaluate the impact of environmental factors on the microbiome (permutation number equals 9,999), calculated alpha diversity using the Chao1 and Shannon indexes, and detected the Spearman correlation coefficients among the species with relative abundance over 0.01%. We kept the relations with coefficients <−0.6 or > 0.6 (adjusted p-value < 0.05) to construct the bacterial interacting matrix (Supplementary Data Sheets 2, 3) and to plot the bacterial co-occurrence networks by applying Gephi (version 0.4.2) and Cytoscape (version 3.8.2) for further analysis (Shannon et al., 2003; Bastian et al., 2009; Otasek et al., 2019). Species with more than 25 correlations were defined as hub species, which indicated their pivotal places in the bacterial co-occurrence networks. We screened and compared these species between different microbiomes.

### Local Stability Analysis

**FIGURE 1:**
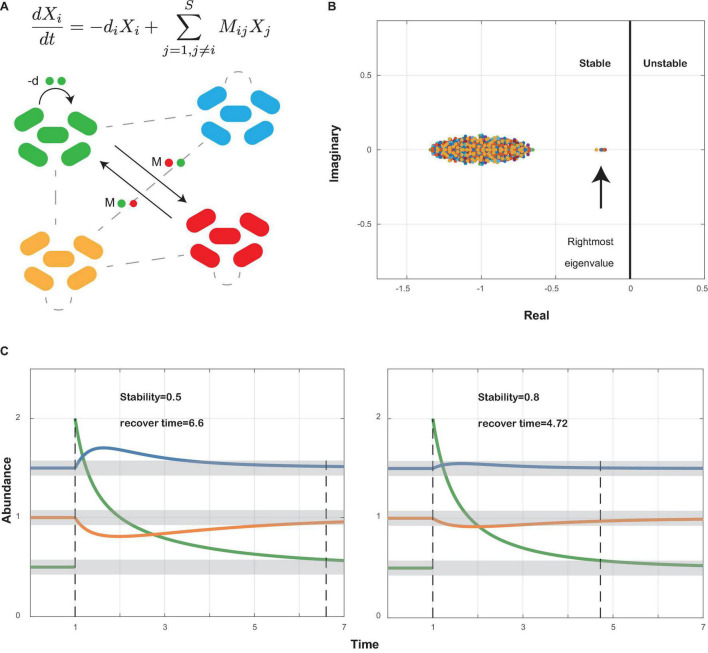
Local stability theory. **(A)** A schematic diagram shows a small community with bacterial species interacting within themselves (−*d*) and with other species (*M*_*ij*_). Ordinary differential equations measure the abundance change of species *i* after perturbation around the equilibrium point. *X*_*i*_, abundance of species *i*; −*d*_*i*_, self-regulating effect of species *i*; *M*_*ij*_, effect of species *j* on species *i*. **(B)** All eigenvalues of community matrix *M* are shown in the complex plane. The community is stable if all eigenvalues have negative real parts. Therefore, the sign of the rightmost eigenvalue decides whether a community is stable or not, and the value of its real part decides how stable the community is: the more negative its real part, the more stable the community (see also Supplementary Information). **(C)** A community will return to its former equilibrium after perturbations if it is stable. A community with higher stability will recover faster than a less stable community.

Local stability measures the tendency of a community to return to its equilibrium after perturbation. The community is stable if it can return to its equilibrium after perturbation. Following the work by May and Allesina (May, 1972, 1973; Allesina and Tang, 2012), we used the community matrix generated from our co-occurrence network (Supplementary Data Sheets 2, 3) to analyze the local stability of oral microbiome (Figure 1A). The community matrix incorporates several structural properties, including the number of interacting species, the connectance, the types and strength of interactions, and the degree distribution. Connectance was defined as the fraction of non-zero off-diagonal elements of the community matrix (May, 1972, 1973), or briefly as the ratio of actual bacterial correlations to all topologically possible correlations. The types of interactions were extracted from our co-occurrence networks illustrated above. The degree of a species referred to the count of its correlations with other species. The local stability theory indicates that a stable system requires that all eigenvalues of the community matrix should have negative real parts (Figure 1B), which means the real part of the rightmost eigenvalue in the complex plane can be used to measure the extent of stability. A more negative real part corresponds to a more stable community, which grants it more robustness when resisting perturbations that tend to alter the abundance of its members (Figure 1C). Based on experimental data, we performed a series of simulations to show the differences in stability among different groups (see also Supplementary Information).

### Statistics

For the Chao1 and Shannon indexes calculated for different groups, we performed the Wilcoxon rank-sum test to check whether significant differences exist between groups. All the Spearman correlation coefficients among the species were adjusted with Benjamini and Hochberg method (adjusted *p* < 0.05). As for the counts of negative and positive correlations, we applied the chi-square test for the detection of significant differences between the health and disease groups.

## Results

### Taxonomical Annotation

After low-quality filtration and host-read removal, a total of 1,926,649,953 sequences were obtained from 64 samples, with an average of 30,103,906 sequences per sample (range from 1,004,522 to 77,090,552). Overall, 310 bacterial species have been identified (see Supplementary Data Sheet 1). The clinical and demographic characteristics of recruited subjects were summarized (Supplementary Table 3). There were no significant differences in mean age and sex distribution among all subjects, and functional time between healthy and diseased implants (*p* > 0.05).

PERMANOVA was performed to evaluate the differences in microbial communities contributed by several factors (Supplementary Figure 1). The results indicated a significant difference between the compositions of supra- and subgingival communities (*R*^2^ = 0.02631, *p* = 0.047). Based on this finding, we therefore analyze and discuss supragingival and subgingival communities separately in the following procedures.

**FIGURE 2:**
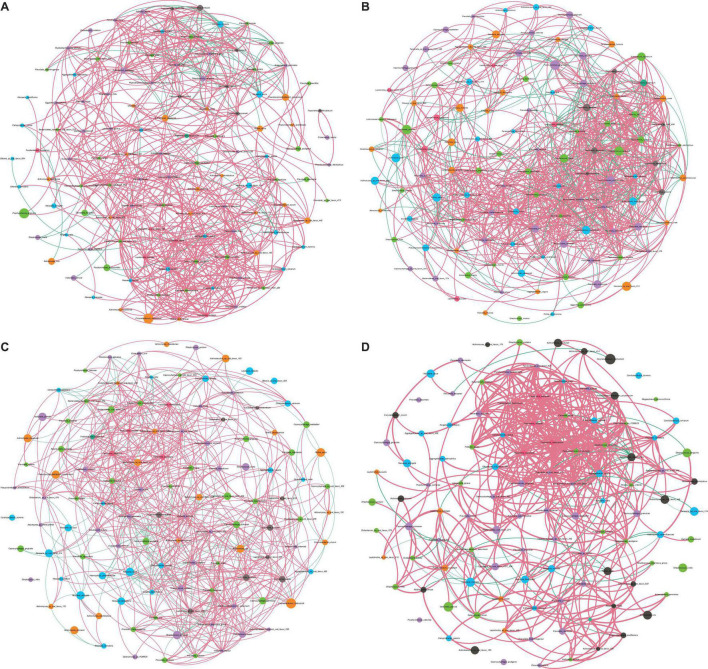
Bacterial co-occurrence networks. **(A)** Network of diseased subgingival microbiome. **(B)** Network of healthy subgingival microbiome. **(C)** Network of diseased supragingival microbiome. **(D)** Network of healthy supragingival microbiome. Species from different phyla were marked in different colors. The larger nodes represented the higher mean relative abundance of the species. We selected those interactions with Spearman correlation coefficient <-0.6 or >0.6 (adjusted *p* < 0.05). Positive and negative correlations are shown in red and green lines, respectively. Thicker lines meant higher absolute values in Spearman coefficient. Generally, the healthy subgingival network was more complex than the diseased subgingival network, while the healthy supragingival network was less complex than the diseased supragingival network.

Using the interacting matrix extracted from our taxonomical annotations (see Materials and Methods), we plotted co-occurrence networks in healthy and diseased sites (Figure 2). In our networks, positive and negative coefficients represented potentially cooperative and competitive interactions between bacterial species, respectively. Overall, subgingival microbiome from periodontitis and peri-implantitis patients exhibited less connected and competitive bacterial networks. On the contrary, supragingival microbiome from the diseased subjects showed more connected and competitive bacterial networks when compared with their healthy controls.

### Structural Properties of Bacterial Co-occurrence Networks

**FIGURE 3:**
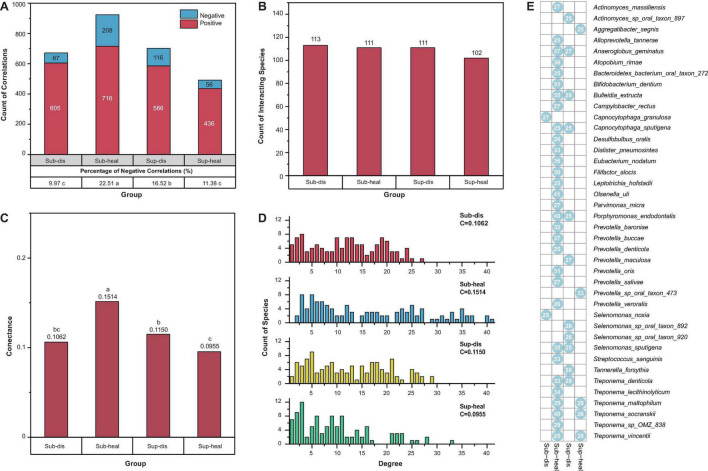
Properties of the community structures in different microbiomes. **(A)** Positive and negative correlations were shown in red and blue, respectively. Positive correlations were predominant in all communities. The percentage of negative correlations in diseased subgingival communities was significantly lower than that in healthy subgingival communities. However, such difference was reversed between diseased and healthy supragingival communities (*p* < 0.05, Pearson chi-square). **(B)** All communities in our study had similar counts of interacting species (*p* > 0.05, Pearson chi-square). **(C)** When associated with periodontitis and peri-implantitis, the subgingival community exhibited a decrease in connectance while the supragingival community exhibited an increase in connectance. Significance of differences was marked in letters. **(D)** Degree distributions of the diseased subgingival, healthy subgingival, diseased supragingival, and healthy supragingival networks are shown in red, blue, yellow, and green bars, respectively. *C* stood for connectance. A conspicuous difference was observed in the degree distribution of healthy subgingival communities as there were significantly more high-degree (degree > 25) species (*p* < 0.05 Pearson chi-square). **(E)** Hub species in the diseased subgingival, healthy subgingival, diseased supragingival, and healthy supragingival microbiome are shown in the heatmap. A blue dot means the species had more than 25 interspecies correlations in the corresponding microbiome. Numbers within the dots showed the counts of correlations of the species.

Besides the proportions of negative and positive interactions, we visualized more structural properties including the numbers of interacting species, the connectance, and the degree distributions of the networks using bar charts (Figures 3A–D), to further dissect the community structure within these networks. In both supra- and subgingival samples, there are similar amounts of interacting species between healthy and diseased microbiome. However, in subgingival microbiome, healthy communities had higher connectance and more high-degree species than diseased communities (*p* < 0.05, Pearson chi-square and Fisher exact test). Besides, the healthy subgingival network had a larger proportion of negative correlations (22.51%, 208 of 924) than the diseased subgingival network (9.97%, 67 of 672) (*p* < 0.05, Pearson chi-square). As for supragingival microbiome, differences were reversed where healthy communities had lower connectance and exhibited a cluster in lower degrees when compared with diseased communities. Also, the healthy supragingival network showed a lower proportion of negative correlations (11.38%, 56 of 492) than the diseased supragingival network (16.52%, 116 of 702) (*p* < 0.05, Pearson chi-square).

Based on the degree distribution, we selected those hub species with more than 25 correlations (degree > 25) in each group. These hub species were the pivotal members in the co-occurrence networks which were highly connected with other species (Figure 3E and Supplementary Table 5). There were more hub species in the healthy subgingival microbiome than the diseased subgingival microbiome (31 in healthy microbiome and 2 in diseased microbiome). Such difference was again reversed in the supragingival group where diseased microbiome had more hub species (5 in healthy microbiome and 11 in diseased microbiome). The results above revealed distinct bacterial co-occurrence networks and community structures in different microbiomes and built the foundation for further stability analysis.

### Alterations in Bacterial Interactions

**FIGURE 4:**
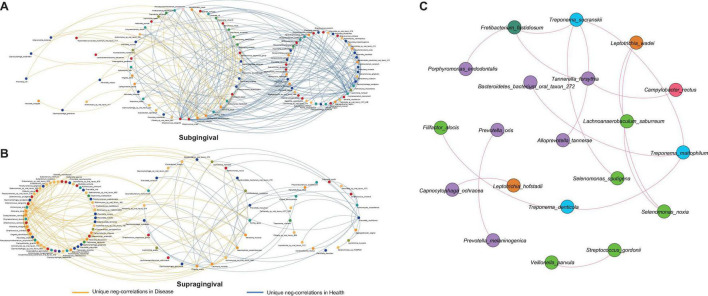
Alterations in negative correlations from health to disease and the shared correlations. **(A)** Unique negative correlations of healthy and diseased subgingival communities. *Streptococcus sanguinis*, *Streptococcus oralis*, *Haemophilus parainfluenzae*, *Rothia aeria*, *Corynebacterium matruchotii*, *Leptotrichia hofstadii*, *Actinomyces massiliensi*s, and *Capnocytophaga sputigena* were the concentrated nodes of negative correlations in health, while *Lautropia mirabilis*, *Actinomyces naeslundii*, and *Capnocytophaga gingivalis* were the concentrated nodes in disease. **(B)** Unique negative correlations of healthy and diseased supragingival communities. *Kingella oralis*, *Lautropia mirabilis*, *Prevotella multiformis*, and *Actinomyces massiliensis* were the concentrated nodes of negative correlations in health, while *Streptococcus sanguinis*, *Neisseria sicca*, and *Capnocytophaga sputigena* were the concentrated nodes of negative correlations in disease. **(C)** The shared correlations of all communities. All shared correlations were positive and were mainly constructed by phyla *Bacteroidetes*, *Firmicutes*, and *Spirochaetes*.

Bacterial interactions are known to have an impact on oral health (Diaz and Valm, 2020), especially the competitive interactions which have been proved essential in preserving the fitness of microbial communities (Stacy et al., 2014). To evaluate how inflammation around teeth and implants would alter such bacterial interactions, we extracted all negative correlations unique to different health conditions for further comparison (Figure 4). As expected, there was a great change in the bacterial competition with the shift from health to disease. Each group had its own distinctive set of unique correlations.

In subgingival microbiome (Figure 4A), *Streptococcus sanguinis* (*l* = 31, number of negative linkages equal 31 with *R* < -0.6 and adjusted *p* < 0.05), *Streptococcus oralis* (*l* = 17), *Haemophilus parainfluenzae* (*l* = 10), *Rothia aeria* (*l* = 12), *Corynebacterium matruchotii* (*l* = 18), *Leptotrichia hofstadii* (*l* = 11), *Actinomyces massiliensi*s (*l* = 22), and *Capnocytophaga sputigena* (*l* = 14) participated in a large number of negative correlations in healthy communities. When inflammation arose, the negative correlations were significantly weakened and those interactions associated with the above species were altered, among which *Corynebacterium matruchotii*, *Leptotrichia hofstadii*, *Actinomyces massiliensi*s, and *Capnocytophaga sputigena* lost all their negative correlations, while *Streptococcus sanguinis* (*l* = 10), *Streptococcus oralis* (*l* = 3), *Haemophilus parainfluenzae* (*l* = 5), and *Rothia aeria* (*l* = 2) established fewer new negative correlations with other species. Instead, in the diseased communities, *Lautropia mirabilis* (*l* = 15), *Actinomyces naeslundii* (*l* = 8), and *Capnocytophaga gingivalis* (*l* = 7) emerged to become the concentrated nodes of negative correlations.

Changes in supragingival microbiome were quite different (Figure 4B), where healthy communities had significantly fewer negative correlations than diseased communities. *Kingella oralis* (*l* = 3), *Lautropia mirabilis* (*l* = 9), *Prevotella multiformis* (*l* = 5), and *Actinomyces massiliensis* (*l* = 15) were the major contributors of negative correlations in healthy communities, while in diseased communities, there were complex sets of negative correlations coming from *Streptococcus sanguinis* (*l* = 14), *Neisseria sicca* (*l* = 10), and *Capnocytophaga sputigena* (*l* = 23).

In contrast with alterations of negative correlations, there were also some correlations shared by all communities despite health conditions or sampling sites (Figure 4C). This shared network was mainly constructed by species from phyla *Bacteroidetes*, *Firmicutes*, and *Spirochaetes*. Different from the unique negative correlations which defined the health status of the microbiome, these shared correlations seemed to be constant and might have formed a fundamental framework for periodontal and peri-implant microbiome.

### Stability Analysis

To compare the stability among different microbial communities, the above structural properties were required for numerical simulations. The number of interacting species, the connectance, and the types of interactions could be drawn directly from our taxonomical annotation and the co-occurrence networks. However, acquiring the strength of interactions would usually require a time-sequence analysis from longitudinal samples according to previous studies (Schloissnig et al., 2013; Stein et al., 2013; Oh et al., 2016). This seemed inapplicable to studying diseased subjects due to ethical reasons, as clinicians were supposed to treat the periodontitis or peri-implantitis rather than observing the diseased status without interference. In this scenario, we introduced a strategy to analyze the stability of microbial communities using cross-sectional samples based on Spearman coefficient (see Materials and Methods, see also Supplementary Information).

**FIGURE 5:**
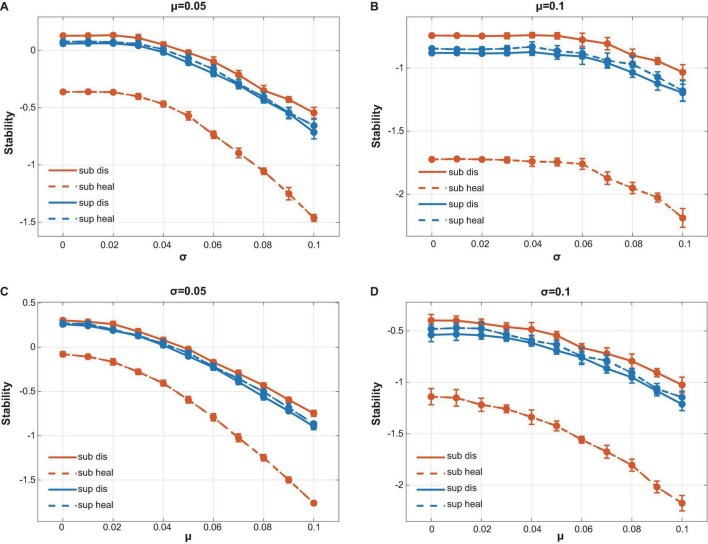
Calculation of local stability. Red lines stood for supragingival communities while blue lines stood for subgingival communities. Healthy and diseased communities are shown in dotted and solid lines, respectively. Connectance, interacting species richness, and bacterial correlations were drawn directly from our interacting matrix. The strength of bacterial interactions was assigned to follow a normal distribution with mean μ and variance σ^2^. By changing the value of μ and σ, we performed a series of calculations to compare the stability of our communities (see also Supplementary Figure 3). **(A)** μ **=** 0.05 with variable σ. **(B)** μ = 0.1 with variable σ. **(C)** σ = 0.05 with variable μ. **(D)** σ = 0.1 with variable μ. All calculations showed the same tendency that the healthy subgingival communities had the worst local stability while the diseased subgingival communities had the highest. However, the stability difference in supragingival communities was not as distinct as that in subgingival communities.

We assigned the strength of interactions following the assumptions by Allesina (Allesina and Tang, 2012) (see Supplementary Information) and mainly focused on comparing the stability among different communities rather than numerically calculating the absolute stability value of a specific community. Stability analysis showed that healthy subgingival communities had the worst stability among four groups while diseased subgingival communities possessed the highest stability (Figure 5). As for the supragingival group, the healthy and diseased supragingival communities showed similar stability in our analysis. We performed a series of simulations using different parameter sets and concluded the same result, which proved its robustness (Figure 5, see also Supplementary Figure 3).

**FIGURE 6:**
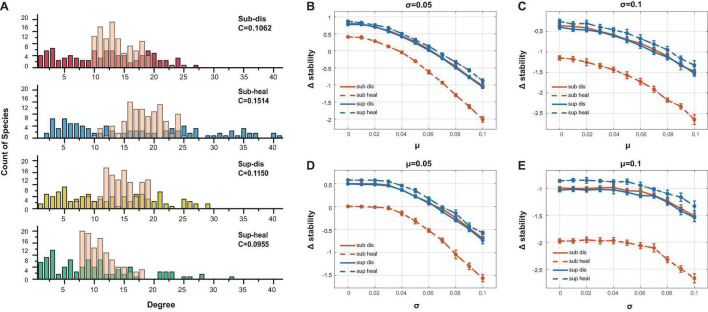
Evaluation of the association between degree distribution and local stability. **(A)** The transparent pink bars showed the degree distribution of the ER networks while the opaque-colored bars showed the degree distribution of the original networks. The major difference was that the ER networks had concentrated degree distributions. **(B)–(E)** The vertical axis showed the stability change after ER randomization (Δ*stability*). Δ*stability* = *sta*_*origin*_−*sta*_*ER*_, in which *sta*_*origin*_ was the stability of the original communities, while *sta*_*ER*_ was the stability of the ER networks. It was clear that all original communities were less stable than their ER counterparts in all parameter sets (different μ and σ as in Figure 5), which indicated that their network structure tended to be destabilizing. The extent of stability decrease in the healthy subgingival group was much more than the other three groups, which meant that the network structure of the healthy subgingival community hampered stability the most.

To figure out why healthy subgingival microbiome was far less stable than the others, we generated unstructured ER (Erdõs–Rényi) networks with the same amount of interacting species, connectance, and the positive–negative ratio of interactions as our original networks. Yet the sole different property was that these unstructured communities were distinguished from the original communities by having concentrated degree distributions (Figure 6A). Using the same method above, we compared the stability differences caused by distinct degree distributions between the original communities and the unstructured communities (Figures 6B–E). All original communities showed decreased stability when compared with their ER network counterparts in most parameter sets, while the healthy subgingival microbiome showed the largest extent of stability decrease. This indicated that the degree distributions of the original communities were somehow destabilizing, among which the degree distribution of the healthy subgingival microbiome tended to hamper stability the most.

## Discussion

### Distinct Structures Between Healthy and Diseased Communities

The oral microbiome is structurally and functionally organized, which means the properties of a microbial community are more than the sum of the components within it (Kuramitsu et al., 2007; Marsh and Zaura, 2017). To fully understand a microbial community, we are supposed to explore the whole structure and the aggregation of all interactions more than focusing on single or pairwise species. In this scenario, we investigated the bacterial co-occurrence networks and the community structures to explore the effect of periodontitis and peri-implantitis on the oral microbiome in a new perspective.

Our study revealed that when inflammation arose around teeth and implants, the subgingival bacterial networks tended to become less connected and less competitive. However, networks in supragingival communities seemed to shift in an opposite direction, with higher connectance and a larger proportion of competitive interactions in the diseased communities than their healthy counterparts.

Bacterial competition has been reported to be beneficial to both competitors involved and might even improve the fitness of the whole microbial community (Stacy et al., 2014), as they form a defensive mechanism in oral microbiome where the colonization of exogenous species was prevented (Marsh and Zaura, 2017). However, our results indicated that inflammation would alter the competition among species in periodontal and peri-implant microbiome. Such alterations could be observed in both supra- and subgingival microbiome and were not just in terms of number or proportion. In fact, the whole community seemed to reestablish a brand-new network with its own distinctive negative correlations and own centers for these correlations. These major changes in the community structure might lead to changes in the keystone compositions of the biofilm and come with the pathologic shift from health to disease (Marsh and Zaura, 2017).

The degree distribution of ecological networks is usually right-skewed with many low-degree vertices and only a small number of high-degree vertices (Girvan and Newman, 2002). Such was the case in our networks where the majority of the species were in low degrees. However, it was still clear that the degree distribution of the healthy subgingival microbiome distinguished itself among groups by having significantly more hub species, which also contributed to hampering the local stability of healthy subgingival community according to our further analysis.

The connectance was another important property of the community structure. Our result showed that when associated with periodontitis and peri-implantitis, the connectance of subgingival microbiome tended to decrease while the connectance of supragingival microbiome tended to increase. Previous studies proved that an ecosystem with higher connectance was more persistent when subjects to colonization–extinction dynamics (Gravel et al., 2011) and was less prone to losing hub species than systems with lower connectance (Kulkarni and De Laender, 2017). However, other studies on the dynamics of complex ecosystems showed that when connectance rose beyond a certain threshold, the local stability of the community would decrease rapidly (Gardner and Ashby, 1970). The healthy subgingival microbiome in our study had a larger number of hub species, which were sensitive to selective loss accordingly. Nonetheless, the high connectance helped prevent these species from losing. As for whether the connectance of our communities had crossed the threshold where local stability began to drop, we suggested that more studies were needed to draw the conclusion. However, we were able to plot the overall outcome of these factors and to compare the stability differences between the healthy subgingival microbiome and the other three groups (see below).

All findings above showed that healthy and diseased oral microbiome had distinct community structures. We addressed that these aberrant changes in bacterial competition, connectance, and degree distribution were crucially associated with the onset and progression of periodontitis and peri-implantitis. Among all communities in our study, we found that the differences between healthy and diseased subgingival microbiomes were most striking and complicated. Future studies should pay more attention to the relationship between community structures and oral infectious diseases, especially the changes in the community structure of subgingival microbiome.

### Association Between Ecological Stability and Health Conditions

Patterns of the bacterial networks in supra- and subgingival microbiome were associated with health and disease. Moreover, the multiple interactions gave the community resilience to environmental perturbations (Marsh and Zaura, 2017). As mentioned above, the stability of a community mainly depends on its community matrix, which incorporates structural properties such as interaction types, connectance, and degree distribution. According to previous studies, competitive interactions tend to increase stability by decreasing diversity within the influence range of the competitors (Coyte et al., 2015), while connectance that reaches beyond a critical level might rapidly destabilize a microbial community (Gardner and Ashby, 1970; May, 1972). Interestingly, in our study, those communities with larger proportions of competitive interactions turned out to have higher connectance too. These communities, or more specifically, healthy subgingival communities and diseased supragingival communities, received antagonistic effects from both stronger competition within species and higher connectance. To plot the outcome of various effects on the stability in our study, we performed a series of simulations following the work of Allesina to compare the stability differences among our communities.

The result showed that healthy subgingival microbiome had the worst local stability among four groups while diseased subgingival microbiome had the highest. This meant that the equilibrium of healthy subgingival microbiome was more delicate and more prone to perturbations. When perturbations reached beyond resilience, equilibrium may break down with changes in microbial composition and shift in the community structure. That could be where dysbiosis happened and be the essence of the initiation of periodontal and peri-implant diseases. On the other hand, the high local stability in diseased subgingival microbiome explained why, if without interventions, the periodontal and peri-implant microbiome could not spontaneously change back to health once infected by periodontitis or peri-implantitis as the diseased equilibrium was very robust.

By comparing the stability between randomly generated ER communities and our original communities, we revealed that the degree distribution of healthy subgingival microbiome tended to be most destabilizing. As healthy subgingival microbiome was characterized by having more hub species, we hereby hypothesized that hub species were in some way a weak point during the breakdown of the current equilibrium, for changes in these highly connected species could trigger a massive alteration in the whole network. This explained why the stability of healthy subgingival microbiome was far lower than other microbiomes. In this scenario, we suggested that more caution should be raised toward these hub species together with their roles during the shift from health to disease.

### Relationship Between Hub Species and Health Conditions

Hub species were those with a large number of interspecies correlations. Whether abundant or not, hub species played roles as “traffic centers” in the bacterial network. In one respect, these species were spatially or functionally related with many others and therefore contributed to the integration of the community. In another respect, they might also be responsible for destabilizing the community as mentioned above. Our study showed that the healthy subgingival microbiome had the highest count of hub species, of which species from genus *Prevotella* and *Treponema* made up a major part. In the diseased subgingival microbiome, there were only two hub species, *Capnocytophaga granulosa* and *Selenomonas noxia*. As for the supragingival microbiome, differences between healthy and diseased networks were not as distinct as subgingival microbiome and seemed to change in an opposite direction where the diseased network had more hub species than the healthy one. The supragingival hub species came from various genus including *Actinomyces*, *Aggregatibacter*, *Anaeroglobus*, *Bulleidia*, *Capnocytophaga*, *Porphyromonas*, *Prevotella*, *Selenomonas*, *Tannerella*, and *Treponema*.

The microbial community is extremely complex and sophisticated which subjects to numerous influences ranging from microbial compositions to environmental and genetic factors. It is difficult to explicitly address the role of a specific species in the community. Although most of the hub species of communities in our study had been proven associated with periodontal and peri-implant destruction (Morita et al., 1991; Ellen and Galimanas, 2000; Takeuchi et al., 2001; Ohishi et al., 2005), we suggested that their pivotal roles in the bacterial network should be treated dialectically, the roles that on the one hand contributed to their pathogenicity, but on the other hand, were also essential in integrating the community network. Future studies should pay more attention to the important roles of these hub species and associate the pivotal places in the network with their pathogenicity.

### Limitations of the Study

One major limitation in this study is that the sample size, although equivalent to other congener studies (Dabdoub et al., 2016; Belstrom et al., 2017; Komatsu et al., 2020), is relatively small to describe the oral microbiome of the whole human population. As the oral microbiome is very individualized (Belibasakis et al., 2019), we suggest that future studies with a larger sample size are needed to further generalize our findings.

The strategy provided in this study is sound and rigorous in theoretical aspect. However, these methods were mainly based on taxonomical annotations. They revealed the phenomena observed from the samples within this study yet could not validate the mechanisms behind the phenomena in biochemistry or molecular view. We appeal that further studies using either *in vitro* models or *in vivo* trials are needed to figure out the detailed mechanisms and provide more clinical implications.

Predicting the stability of microbial community usually requires a time-sequence analysis from longitudinal samples, as longitudinal studies offer control for confounding factors including age, gender, diet and so on. Although cross-sectional samples can also provide prediction on community stability following our strategy, it can be less powerful than longitudinal ones (Knight et al., 2018).

## Conclusion

In conclusion, we revealed distinct community structures in healthy and diseased microbial communities around teeth and implants. By extracting the bacterial correlation networks, we found that the subgingival microbiome tended to become less connective and competitive when inflammation arises. In contrast, the supragingival microbiome tended to become more connective and competitive. We also observed a great change in competitive interspecies correlations between healthy and diseased microbiome. These alterations contributed crucially to the shift from health to disease and were highly associated with periodontal and peri-implant microbiome dysbiosis in the aspect of community structures. Besides, by applying dynamic models on these microbial communities, we concluded that the healthy subgingival community was far less stable than the inflamed subgingival community. We also managed to prove that it was those highly connected species in the network that contributed to destabilizing the biofilm. Our results suggested these hub species should also be given more concern in future studies. Preserving these species and maintaining their normal functionalities might be of much meaning in preventing periodontal and peri-implant diseases. Combining the above findings, we revealed that microbiome dysbiosis in the periodontium was not limited to the changes in bacterial compositions. With durative perturbations from microbial pathogens, the former equilibrium broke down and the microbiomes formed new bacterial networks with distinct interspecies correlations and community structures. During this progress, the subgingival biofilm established a more stable and stubborn community with even higher resilience.

## Data Availability Statement

The original contributions presented in the study are included in the article/Supplementary Material, further inquiries can be directed to the corresponding author/s.

## Ethics Statement

The studies involving human participants were reviewed and approved by Ethics Committee of College of Stomatology, Xi’an Jiaotong University. The patients/participants provided their written informed consent to participate in this study. Written informed consent was obtained from the individual(s) for the publication of any potentially identifiable images or data included in this article.

## Author Contributions

YZ designed the details of the study, conducted the statistical analysis, interpreted the analysis results, and wrote this manuscript. YL performed the bioinformatics analyses, interpreted the analysis results, and revised the manuscript. YY conducted mathematical simulations and interpreted the results. YW helped perform statistical analysis and revised the manuscript. XC and YX helped with the collection of samples and the revision of the manuscript. YJ revised the manuscript and performed statistical analysis. QZ and SCL supervised the whole project and polished the manuscript. All authors reviewed and approved the manuscript.

## Conflict of Interest

The authors declare that the research was conducted in the absence of any commercial or financial relationships that could be construed as a potential conflict of interest.

## Publisher’s Note

All claims expressed in this article are solely those of the authors and do not necessarily represent those of their affiliated organizations, or those of the publisher, the editors and the reviewers. Any product that may be evaluated in this article, or claim that may be made by its manufacturer, is not guaranteed or endorsed by the publisher.
