# Supplementary material for: Periodontal and Peri-Implant Microbiome Dysbiosis Is Associated With Alterations in the Microbial Community Structure and Local Stability
Source: Front Microbiol. 2022 Jan 25;12:785191. doi: 10.3389/fmicb.2021.785191 (PMC8821947; doi:10.3389/fmicb.2021.785191)
Supplement: Supplementary file 1 [file Presentation_1.pdf]

Supplementary Information

Examiner calibration

Before the study, intra-examiner calibration had been performed by measuring the PD and radiographic bone loss of 20 teeth and 10 implants not included in the study three times within a day. Each measurement had a minimum intermission of 1 hour. The results were taken as consistent when the error among three measurements was no more than 1mm. The percentage of consistency should be no less than 90% to pass the intra-examiner calibration. The total percentage of consistency on this examiner turned out to be 93.33%.

Sampling sites selection

Selection of sampling sites followed these criteria: 1) For periodontal health subjects, four first molars were selected for sampling; 2) For subjects with periodontitis, the plaque was sampled from the teeth with deepest probing depth per quadrant; 3) For subjects with implants, samples were taken from the specific implants which met our inclusion criteria in Table 1. Detailed information of all samples can be seen in Supplementary Tables 1 and 2.

PERMANOVA and core microbiome computation

As illustrated in the main text, after annotations at species level, we performed PERMANOVA to evaluate the impact of different factors on the compositions of the microbiome (Supplementary Figure 1A). The results indicated that supragingival and subgingival microbiome were significantly different. This was confirmed by PCoA on the beta diversity of supra- and subgingival microbiome (Supplementary Figure 1B). Then we compared the alpha diversity using Chao1 and Shannon indices (Supplementary Figure 1C). The result showed that supragingival microbiome had significantly higher Chao1 index yet similar Shannon index when compared with subgingival microbiome. This indicated that the supragingival communities had higher species richness, however, some of the species were either too high or too little in abundance which resulted in poorer evenness in comparison. We then computed the core microbiome in supra- and subgingival communities (Supplementary Figure 1D). Core species represented those bacteria members shared by at least 80% of individuals in either supra- or subgingival microbiome with a minimum relative abundance of 0.1%. Detailed lists of core species were presented in Supplementary Table 4.

We also compared the relative abundance of these core species between healthy and diseased

individuals (Supplementary Figure 2). There were no significant differences in relative abundance in any of the core species, indicating that the core components of supragingival (or subgingival) communities were in a way constant and did not change with the shift of health conditions. We therefore took a further look into the community structure to figure out what factors differentiate the healthy and diseased microbiome as shown in the main text.

Stability analysis

Here we briefly review local asymptotic stability (henceforth, stability) and show how to analyze the stability of the oral microbiome through experimental data and numerical simulation.

1. Dynamical framework

Following May's assumptions, we consider the microbial community- which consists of S interacting species- as an autonomous system. The dynamical behavior of this system can be described by a set of ordinary differential equations:

$$\frac{dX_i}{dt} = f_i(\boldsymbol{X}(t))$$

where $X_i(t)$ represents, for example, the abundance of population i at time t, and f_i is the function expressing the growth rate of population i, which depends on the abundance of all populations. The point $\boldsymbol{X}^* > 0$ is a feasible equilibrium if $f_i(\boldsymbol{X}^*) = 0$ for all i.

Around the equilibrium, the trajectories can be described by considering a linearized system. Suppose the system is resting at the equilibrium $\boldsymbol{X}^*$, and that a sufficiently small perturbation is applied at time zero, $\boldsymbol{X}(0) = \boldsymbol{X}^* - \boldsymbol{x}(0)$. Then, by Taylor expansion:

$$\frac{d\boldsymbol{x}(t)}{dt} \approx \boldsymbol{J}(\boldsymbol{X}^*)\boldsymbol{x}(t)$$

where $\boldsymbol{J}$ is the Jacobian matrix of the system, $J_{ij} = \frac{\partial f_i}{\partial x_i}$. The so-called "community matrix $\boldsymbol{M}$" is the Jacobian evaluated at $\boldsymbol{X}^*$, and therefore:

$$\frac{d\boldsymbol{x}(t)}{dt} \approx \boldsymbol{M}\boldsymbol{x}(t)$$

which is a system of homogeneous linear differential equations with constant coefficients. This system has solution:

$$\boldsymbol{x}(t) = e^{\boldsymbol{M}t}\boldsymbol{x}(0)$$

Moreover, if $\boldsymbol{M}$ is diagonalizable, it can be decomposed as $\boldsymbol{P}^{-1}\boldsymbol{\Lambda}\boldsymbol{P}$ where $\boldsymbol{\Lambda}$ is a diagonal matrix whose diagonal coefficients are the eigenvalues of $\boldsymbol{M}$, and $\boldsymbol{P}$ is a matrix whose columns are the corresponding right eigenvectors. In this case, the solution becomes:

$$\boldsymbol{x}(t) = \boldsymbol{P}^{-1}e^{\boldsymbol{\Lambda}t}\boldsymbol{P}\boldsymbol{x}(0)$$

If all the eigenvalues of $\boldsymbol{M}$ have negative real part, the small perturbation $\boldsymbol{x}(t)$ will eventually decay to zero. Thus, if we order the eigenvalues according to their real part, $R_e(\lambda_{\boldsymbol{M},1}) > \cdots > R_e(\lambda_{\boldsymbol{M},S})$, stability is exclusively determined by $R_e(\lambda_{\boldsymbol{M},1})$. If $R_e(\lambda_{\boldsymbol{M},1}) < 0$, the equilibrium is stable, and if $R_e(\lambda_{\boldsymbol{M},1}) > 0$, the equilibrium is unstable.

In fact, $R_e(\lambda_{\boldsymbol{M},1})$ describes the asymptotic decay rate of the system after perturbation. Thus, $R_e(\lambda_{\boldsymbol{M},1})$ is often used as a measure of the system's stability. Following previous work, we here define the system's stability as $-R_e(\lambda_{\boldsymbol{M},1})$.

2. Stability analysis using experimental data and numerical simulation

What mentioned above shows that the key to stability analysis is the construction of community matrix $\boldsymbol{M}$, $\boldsymbol{M}$ can be constructed by the following two steps. Firstly, we generated the adjacency matrix $\boldsymbol{K}$ from our taxonomical data. $K_{ij} > 0$ meant species i received a positive effect from species j, $K_{ij} < 0$ meant species i received a positive effect from species j while $K_{ij} = 0$ meant species j had no effect on species i. Secondly, we assigned the coefficients of $\boldsymbol{M}$ as follows:

$$\begin{cases} K_{ij} > 0 \rightarrow M_{ij} = |Z| \\ K_{ij} < 0 \rightarrow M_{ij} = -|Z| \\ K_{ii} = -d \end{cases}$$

where Z was a random variable obeying normal distribution $N(\mu, \sigma^2)$.

We then performed a series of numerical simulations by changing μ and σ. For each parameter combination, we performed 50 simulations (Figure 5 and Supplementary Figure 3). The results showed that healthy subgingival communities possessed the worst stability while the diseased subgingival communities possessed the highest stability.

Supplementary Tables

Supplementary Table 1. Detailed information of samples from teeth.

Subject Code	Sample Code		Age (years)	Sex	PD (mm)	RBL (mm)
	Supragingival	Subgingival				
**HT-1**	A2	A3	32	Male	2	0
**HT-2**	D2	B2	46	Male	2	1
**HT-3**	E2	E3	52	Female	3	1
**HT-4**	H-H-up	H-H-dw	38	Male	2	0
**HT-5**	H-I-up	H-I-dw	50	Female	3	0
**HT-6**	H-G-up	H-G-dw	29	Female	1	0
**HT-7**	H-K-up	H-K-dw	44	Male	3	1
**HT-8**	H-O-up	H-O-dw	37	Male	3	0
**HT-9**	H-N-up	H-N-dw	36	Male	2	0
**HT-10**	/	C2	40	Female	2	0
**DT-1**	CP-a-up	CP-a-dw	46	Male	6	3
**DT-2**	CP-c-up	/	62	Male	5	3
**DT-3**	CP-d-up	CP-d-dw	41	Female	6	4
**DT-4**	CP-f-up	CP-f-dw	50	Male	5	3
**DT-5**	CP-g-up	CP-g-dw	46	Male	7	4
**DT-6**	CP-h-up	CP-h-dw	61	Male	4	3
**DT-7**	CP-m-up	CP-m-dw	52	Male	5	4
**DT-8**	CP-o-up	CP-o-dw	36	Female	5	3
**DT-9**	CP-q-up	CP-q-dw	48	Male	7	5
**DT-10**	CP-t-up	CP-t-dw	68	Female	6	4

HT and DT stand for healthy teeth and teeth with periodontitis, respectively. Note that the supragingival sample from subject HT-1 was discarded due to contamination during transportation. The subgingival sample from subject DT-2 failed to pass quality control after filtration and was therefore discarded.

Supplementary Table 2. Detailed information of samples from dental implants.

Subject Code	Sample Code		Age (years)	Sex	PD (mm)	RBL (mm)	Functional Time (years)	Implant Location
	Supragingival	Subgingival						
**HI-1**	HIU-182	HID-184	51	Female	4	1	4	Posterior
**HI-2**	HIU-186	HID-188	51	Female	3	0	2	Posterior
**HI-3**	HIU-222	HID-224	63	Female	4	0	4	Posterior
**HI-4**	HIU-232	HID-234	68	Female	4	1	3	Posterior
**HI-5**	HIU-242	HID-244	44	Male	4	0	5	Posterior
**HI-6**	HIU-252	HID-254	48	Male	4	0	4	Posterior
**HI-7**	HIU-256	HID-258	37	Male	3	0	4	Posterior
**HI-8**	HIU-A2	HID-B2	60	Female	3	0	3	Posterior
**HI-9**	HIU-B1	HID-B3	33	Female	2	1	4	Posterior
**DI-1**	IIU-31	IID-33	29	Male	7	3	3	Posterior
**DI-2**	IIU-42	IID-44	65	Female	8	5	4	Anterior
**DI-3**	IIU-82	IID-84	41	Female	6	3	3	Posterior
**DI-4**	/	IID-194	39	Female	6	3	5	Posterior
**DI-5**	/	IID-264	56	Male	7	4	9	Anterior

HI and DI stand for healthy implants and implants with peri-implantitis, respectively. Note that the supragingival samples from subjects DI-4 and DI-5 were discarded due to contamination during transportation.

Supplementary Table 3. Summary of clinical and demographic characteristics of all samples.

Group Category	Mean Age (years)	Sex Distribution (Female%)	Average PD (mm)	Average RBL (mm)	Functional Time (years)	Implant Location		Implant System		
						Anterior	Posterior	Osstem	Bego	ITI
**Group T**	**45.70**	**35.00**	**3.95**	**1.95**	**/**	**/**	**/**	**/**	**/**	**/**
**HT**	40.40	40.00	2.30	0.30	/	/	/	/	/	/
**DT**	51.00	30.00	5.60	3.60	/	/	/	/	/	/
**Group I**	**48.93**	**64.29**	**4.64**	**1.50**	**4.07**	**2**	**12**	**4**	**7**	**3**
**HI**	50.56	66.67	3.44	0.33	3.67	0	9	1	5	3
**DI**	46.00	60.00	6.80	3.60	4.80	2	3	3	2	0

Supplementary Table 4. List of core species in supra- and subgingival microbiome.

Subgingival Core Microbiome	Supragingival Core Microbiome
Tannerella_forsythia	
Streptococcus_oralis	Streptococcus_oralis
Actinomyces_oris	Actinomyces_oris
Streptococcus_sanguinis	Streptococcus_sanguinis
Fusobacterium_nucleatum	Fusobacterium_nucleatum
Actinomyces_sp_oral_taxon_414	Actinomyces_sp_oral_taxon_414
Veillonella_parvula	Veillonella_parvula
Lautropia_mirabilis	Lautropia_mirabilis
Actinomyces_naeslundii	Actinomyces_naeslundii
Corynebacterium_matruchotii	Corynebacterium_matruchotii
Pseudopropionibacterium_propionicum	Pseudopropionibacterium_propionicum
Capnocytophaga_gingivalis	Capnocytophaga_gingivalis
Capnocytophaga_sputigena	Capnocytophaga_sputigena
	Campylobacter_gracilis
	Capnocytophaga_leadbetteri
	Capnocytophaga_ochracea
	Neisseria_sicca
	Tannerella_sp_oral_taxon_HOT_286
	Streptococcus_cristatus
	Capnocytophaga_granulosa
	Neisseria_elongata
	Cardiobacterium_valvarum

Supplementary Table 5. List of hub species.

Subgingival Microbiome	Supragingival Microbiome
Sub-heal	Sup-heal
Streptococcus_sanguinis	Aggregatibacter_segnis
Porphyromonas_endodontalis	Treponema_socranskii
Alloprevotella_tannerae	Treponema_maltophilum
Prevotella_denticola	Treponema_vincentii
Actinomyces_massiliensis	Prevotella_sp_oral_taxon_473
Prevotella_oris	Sup-dis
Capnocytophaga_sputigena	Capnocytophaga_sputigena
Treponema_denticola	Tannerella_forsythia
Filifactor_alocis	Treponema_denticola
Eubacterium_nodatum	Porphyromonas_endodontalis
Desulfobulbus_oralis	Selenomonas_sputigena
Anaeroglobus_geminatus	Actinomyces_sp_oral_taxon_897
Treponema_socranskii	Anaeroglobus_geminatus
Atopobium_rimae	Prevotella_maculosa
Parvimonas_micra	Selenomonas_sp_oral_taxon_892
Treponema_vincentii	Bulleidia_extructa
Leptotrichia_hofstadii	Selenomonas_sp_oral_taxon_920
Treponema_maltophilum	
Prevotella_baroniae	
Selenomonas_sputigena	
Prevotella_buccae	
Campylobacter_rectus	
Olsenella_uli	
Bifidobacterium_dentium	
Bacteroidetes_bacterium_oral_taxon_272	
Bulleidia_extructa	
Dialister_pneumosintes	
Prevotella_veroralis	
Treponema_sp_OMZ_838	
Treponema_lecithinolyticum	
Prevotella_salivae	
Sub-dis	
Capnocytophaga_granulosa	
Selenomonas_noxia	

Supplementary Figures

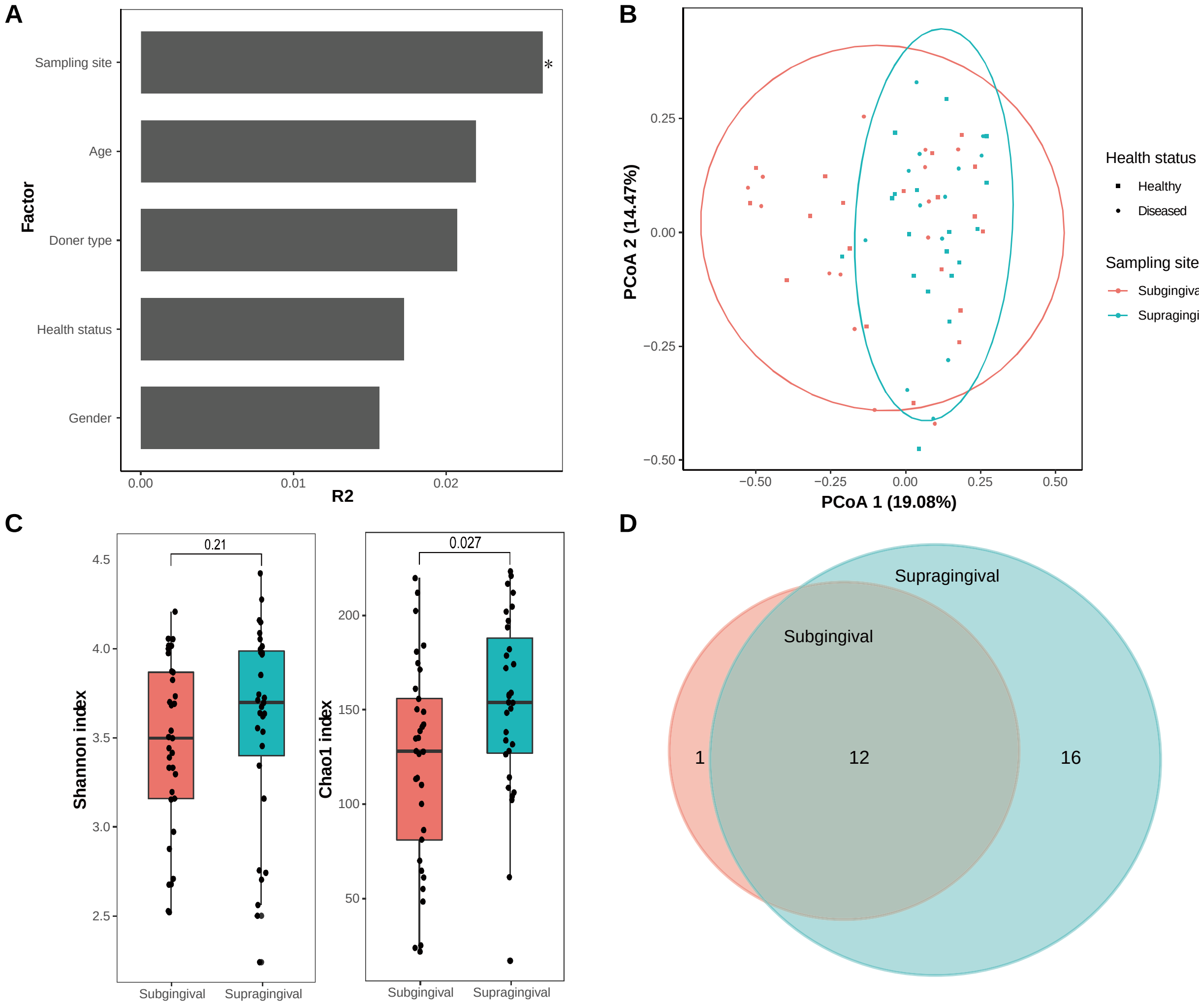

Supplementary Figure 1. Comparison between supragingival and subgingival microbiome. (A) PERMANOVA showed that different sampling sites had distinct microbial communities. **(B)** Principal coordinate analysis showed a difference between supragingival and subgingival communities in terms of beta diversity. The green and red ellipses indicated the 95% confidence regions of the supragingival and subgingival microbiome, respectively. **(C)** Alpha diversity was analyzed using Shannon and Chao1 indices. No significant difference was observed in Shannon index. Chao1 index of supragingival samples was significantly higher than that of subgingival samples. This meant that the supragingival microbiome had higher species richness but poorer evenness. **(D)** Vann diagram showed there were 28 and 13 core species in supragingival and subgingival microbiome, respectively, notably 12 species were shared by both cores.

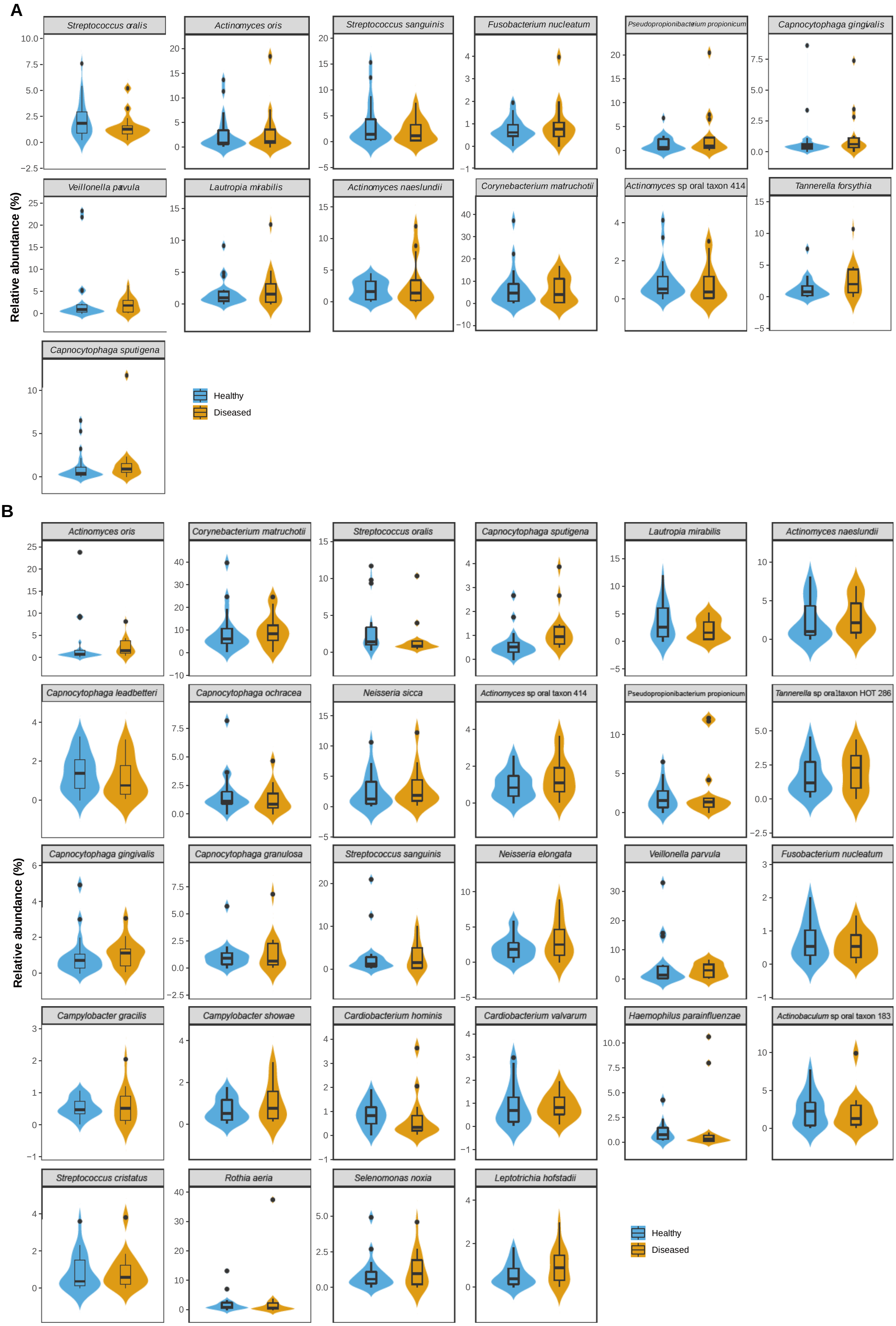
A
Streptococcus oralis
Actinomyces oris
Streptococcus sanguinis
Fusobacterium nucleatum
Pseudopropionibacterium propionicum
Capnocytophaga gingivalis
Veillonella parvula
Lautropia mirabilis
Actinomyces naeslundii
Corynebacterium matruchotii
Actinomyces sp oral taxon 414
Tannerella forsythia
Capnocytophaga sputigena
Relative abundance (%)
Healthy
Diseased
B
Actinomyces oris
Corynebacterium matruchotii
Streptococcus oralis
Capnocytophaga sputigena
Lautropia mirabilis
Actinomyces naeslundii
Capnocytophaga leadbetteri
Capnocytophaga ochracea
Neisseria sicca
Actinomyces sp oral taxon 414
Pseudopropionibacterium propionicum
Tannerella sp oral taxon HOT 286
Capnocytophaga gingivalis
Capnocytophaga granulosa
Streptococcus sanguinis
Neisseria elongata
Veillonella parvula
Fusobacterium nucleatum
Campylobacter gracilis
Campylobacter showae
Cardiobacterium hominis
Cardiobacterium valvarum
Haemophilus parainfluenzae
Actinobaculum sp oral taxon 183
Streptococcus cristatus
Rothia aeria
Selenomonas noxia
Leptotrichia hofstadii
Relative abundance (%)
Healthy
Diseased

Supplementary Figure 2. Comparison of relative abundance of the core species between healthy and diseased microbiome. **(A)** 13 subgingival core species **(B)** 28 supragingival core species. Blue violins and boxes represented healthy microbiome while yellow violins and boxes represented diseased microbiome. No significant differences were detected in the relative abundance of these core species. This indicates the core components of the supra- and subgingival microbiome did not change with the shift of health conditions.

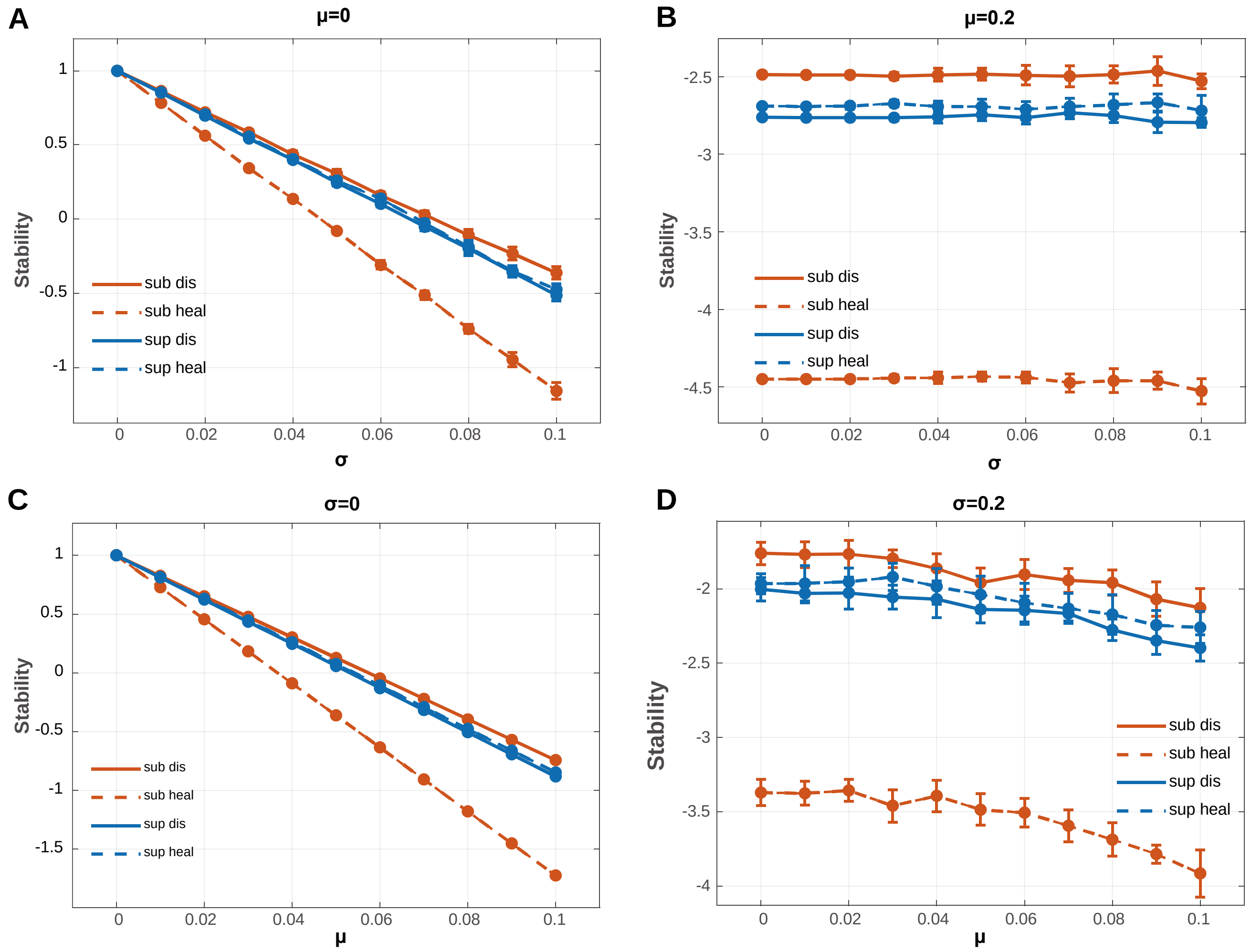

Supplementary Figure 3. Other parameter sets of calculating local stability. Other parameter sets we used to compare stability among communities were shown. All these simulations showed the same result as in Figure 5, which proved the robustness of our findings.
